# A Hospital-based Observational Study of Type 2 Diabetic Subjects from Gujarat, India

**DOI:** 10.3329/jhpn.v29i3.7874

**Published:** 2011-06

**Authors:** Mayur Patel, Ina M. Patel, Yash M. Patel, Suresh K. Rathi

**Affiliations:** ^1^All India Institute of Diabetes and Research, Narainpura, Ahmedabad 380013, Gujarat, India; ^2^S.B.K.S. Medical Institute and Research Centre, Piparia, Vadodara 391760, Gujarat, India

**Keywords:** Type 2 diabetes mellitus, Obesity, Polyuria, Glycaemic status, Dyslipidaemia, India

## Abstract

The aim of this observational study was to describe the profile of subjects with type 2 diabetes mellitus from Gujarat, India. The study was performed with newly-diagnosed 622 type 2 diabetic subjects who attended the Department of Diabetology, All India Institute of Diabetes and Research and Yash Diabetes Specialties Centre (Swasthya), Ahmedabad, during August 2006–January 2009. The subjects completed an interviewer-administered questionnaire. The questionnaire included variables, such as sociodemographic factors, presenting symptoms, risk profile (hypertension, obesity, dyslipidaemia, and glycaemic status), family history of diabetes, physical activity, and behavioural profile. Blood pressure, body mass index (BMI), glycosylated haemoglobin levels, and fasting lipid profile were measured. Descriptive and bivariate analyses were carried out using the SPSS software (version 11.5). In total, 622 type 2 diabetes mellitus (T2DM) cases with mean age of 47.7±10.9 years were studied. Of the 622 subjects, 384 (62%) were male. The majority (68%) of the T2DM subjects were obese, and 67% had a positive family history of diabetes. Renal dysfunctions and vision impairment were, respectively, found in 10% (n=62) and 9% (n=57) of the 622 T2DM subjects. The mean HbA1c level was 9.02±1.67%, and good glycaemic control (HbA1c level <7%) was achieved only in 7.4% of the T2DM subjects. Results of chi-square analysis showed that higher BMI (≥25 kg/m^2^) was significantly associated with hypertension among the T2DM subjects (p<0.01). There were significant differences (p<0.05) between male and female subjects with respect to mean age, BMI, waist and hip-circumference, and mean low-density lipoprotein (LDL) level. The results revealed that many factors, such as obesity, fami-ly history of diabetes, dyslipidaemia, uncontrolled glycaemic status, sedentary lifestyles, and hypertension were prevalent among the T2DM subjects. The characterization of this risk profile will contribute to designing more effective and specific strategies for screening and controlling T2DM in Gujarat, India.

## INTRODUCTION

Type 2 diabetes mellitus (T2DM) is a chronic, debilitating disease characterized by insulin resistance, impaired insulin secretion, and hyperglycaemia. It is the most prevalent metabolic condition and one of the major health and socioeconomic problems worldwide (1-3). It represents more than 90% of the whole diabetic population in the world (4) and is responsible for 9% of global mortality corresponding to four million deaths per year. Since the beginning is insidious, there is an average delay of 3-5 years in diagnosis. By the time the condition is diagnosed, minimal changes responsible for microvascular and macrovascular complications are already present. This issue is further compounded by untreated diabetes because of ignorance or inaccessibility to treatment. Untreated diabetes may result in limb amputation, blindness, kidney failure, and neuropathy. T2DM is also associated with four-fold increased risk of cardiovascular events and is a risk factor for doubling the risk of cardiovascular death (5-7).

The prevalence of T2DM has been rising worldwide, and globally, more than 180 million people are suffering from it. In particular, epidemics of this disorder have taken place in developing societies (8-9). Global diabetic load mostly comes from India and China where more than 75% of diabetic subjects will live in 2025 (4). India, like many other developing countries, has witnessed a rapid epidemiological transition in the last two decades. Coupled with this, there has been a dramatic improvement of the Indian economy in terms of per-capita income. These dramatic changes have had a great impact on urbanization and lifestyle of the Indians. As a result, diabetes mellitus has become the main public-health problem and amenable to change through early recognition at the individual level and surveillance at the population level. Results of studies showed that India is facing three-fold rise in the prevalence of diabetes in urban (5-15%) and in rural (2-6%) areas (10-11). India tops in the world with the largest number of diabetic subjects (31.7 million cases of T2DM) (3,12). This is further compounded by the epidemic of obesity and doubling the cost of diabetes management (13). Therefore, prevention is important from monetary and from lifestyle modification point of view. Increasingawareness of risk factors and how to prevent these should be emphasized in the population (14). Apart from this, the lifestyle modifications (physical exercise, diet control, etc.) are appropriate measures in the prevention of diabetes. Furthermore, to control and prevent the T2DM epidemic, it must be approached in an appropriate, socioeconomically and culturally-relevant manner but very little data are available from Gujarat to support this, and for the prevention of diabetes, it is also vital to know the profile of diabetics.

This paper reports the profile of type 2 diabetic subjects from Gujarat, India.

## MATERIALS AND METHODS

### Study setting

A hospital-based observational study was conducted during August 2006–January 2009 in Ahmedabad district of Gujarat state, India. Ahmedabad is the largest district of Gujarat with an approximate population of five million. The city is famous for medical tourism because of its wide network of cost-effective tertiary-care hospitals catering to the need of population of not only from Gujarat but also from neighbouring states and even from abroad.

### Study population

The study population comprised diabetic subjects. To be included in this study, it was a must for subjects to be newly diagnosed for diabetes mellitus (diagnosed within the last 6 months), attending the Department of Diabetology, All India Institute of Diabetes and Research and Yash Diabetes Specialties Centre (Swasthya) during the study period for the first time and willing to participate in the study.

### Sample-size

A sample-size of 660 was decided on the basis of review of data of the Department of Diabetology which revealed that at least 100 subjects (newly-diagnosed) were presented every month. Hence, there would be 3,000 subjects during the study period. Of the 3,000 newly-diagnosed cases, at least 20% were selected for this study. The calculated minimum sample-size was inflated by 10% to account for anticipated drop-outs.

### Procedure for data collection

After satisfying the case definition and obtaining informed consent (written consent from literate subjects and verbally informed consent from illiterate subjects), 709 subjects were enrolled through simple random-sampling method. After explaining the details of the study, a comprehensive case history was recorded on a semi-structured, close-ended proforma. Basic data on age, sex, education, occupation, smoking and tobacco-chewing status, alcohol consumption, diet, and physical activity were collected from all the subjects. All the subjects were also interviewed regarding history of hypertension and other co-morbid conditions. A general physical examination was performed.

For analysis, the current smokers and ex-smokers were categorized in the ‘ever-smoker’ group. imilarly, the current tobacco chewers and ex-tobacco chewers were categorized in the ‘ever-tobacco chewer’ group. Both ever-smoker group and ever-tobacco chewer group were considered tobacco users. Current alcohol users were defined as subjects who have consumed alcohol at least once in the last one month. Mode of onset of diabetes was defined based on the duration of symptoms of diabetes present in the subjects and categorized as acute (symptoms present for up to one month), sub-acute (symptoms present from one to three months), and insidious (symptoms present from three to six months). The main occupational level was divided into three categories: low (e.g. skilled workers, household workers, and retired persons), medium (e.g. desk-jobs), and high (e.g. professionals and businessmen). Physical activity was categorized as sedentary (sitting, standing, and driving for most of the day, cooking, light cleaning, light yard work, slow walking, and other major activities that involve sitting), moderate (an occupation that includes lifting, lots of walking, or other activities that keep you moving for several hours qualified as moderately active), and heavy (heavy manual labour, a very active lifestyle, dancer, or very active sports played for several hours almost daily, an elite athlete in training, or an extremely active lifestyle―both at work and at play and sport or activi-ty last for several hours, almost daily).

### Anthropometric, clinical and biochemical measurements

All anthropometric measurements were recorded using standardized procedures. The study subjects also underwent various clinical tests, such as urinalysis (first-morning urine sample for evaluation of micro-albuminuria), blood tests for complete haemogram, plasma glucose, glycosylated haemoglobin (HbA1c), renal function tests, and lipid levels. Blood samples were collected after ensuring 12 hours of overnight fasting. Total cholesterol (TC), triglycerides (TG), and high-density lipoprotein-cholesterol (HDL-C) levels were estimated in serum. Low-density lipoprotein-cholesterol (LDL-C) was calculated using the Friedewald formula [LDL-C (mg/dL)=non-HDL-C-TG/5] (15).

Blood pressure was recorded after the subjects had rested for at least five minutes. Two readings were taken five minutes apart, and mean of the two was considered the actual blood pressure. Hypertension was diagnosed based on drug treatment for hypertension or if the blood pressure was >130/80 mmHg according to the Joint National Committee-7 (JNC-VII) criteria (16-17). The diagnosis of diabetes mellitus was made using the criteria established by the American Diabetes Association (18), i.e. a medical record indicating either a fasting plasma glucose (FPG) level of >7.0 mmol/L or ≥126 mg/dL after a minimum 12-hour fasting, or two-hour post glucose level (oral glucose tolerance test) of >11.1 mmol/L or ≥200 mg/dL on more than one occasion, with symptoms of diabetes. In the absence of information from the medical records, confirmation was done on self-reported case by establishing the criteria of regular treatment with anti-diabetic drugs or by performing a two-hour oral glucose tolerance test (OGTT). Impaired glucose tolerance (IGT) was defined as the FPG level of 100 mg/dL (5.6 mmol/L) but <126 mg/dL (7.0 mmol/L) or two-hour OGTT of ≥140 mg/dL (7.8 mmol/L) but <200 mg/dL (11.1 mmol/L).

The guidelines of the National Cholesterol Education Programme were used for defining dyslipidaemia (19). It was defined by presence of one or more than one abnormal serum lipid concentration, such as hypercholesterolaemia, high LDL-C, hypertriglyceridaemia, and low HDL-C. Body mass index (BMI) values were defined according to the recommendations of the Indian Council of Medical Research for Indians. A study subject was considered to be obese if BMI was ≥25 kg/m^2^ and overweight when BMI was 23-24.9 kg/m^2^ (20). The criterion for glycaemic status was <7% (good control), 7-8% (sub-optimal control), 8-9% (inadequate control), and >9% (uncontrolled) (21).

### Analysis of data

Data were processed in Excel-sheet and analyzed using the SPSS software (version 11.5). Quantitative variables were summarized using mean and standard deviation while categorical variables were tabulated using frequencies and percentages. Student's *t-*test was used for testing the significance of differences between the mean values of two continuous variables. Chi-square test was carried out to test the difference between two or more groups. The probability (p) level of less than 0.05 was considered significant.

### Ehical approval

The Institutional Review Board of the All India Institute of Diabetes and Research approved the study protocol.

## RESULTS

A sample of 709 diabetic subjects was enrolled. Of the 709 subjects, 622 (88%) had T2DM. Hence, further analysis was performed on the 622 T2DM subjects.

### Sociodemographic characteristics

Sociodemographic characteristics of the study subjects are shown in Table 1. The type 2 diabetic subjects were evenly distributed in four quartiles of age with mean of 47.70±10.94 years. Of the study samples (n=622), 384 (62%) were male, and 238 (38%) were female. Eighty-four percent (n=521) of the subjects were following religion of Hinduism, and most (99%) study subjects were literate. Thirty-seven percent of the subjects had high level of occupation.

**Table 1.** Sociodemographic characteristics of type 2 diabetic subjects from Gujarat, India

Characteristics	No. (n=622)	%*
Age (years) (mean±SD)	47.70±10.94	
Up to 40	164	27
41-48	170	27
49-55	150	24
>55	138	22
Sex		
Male	384	62
Female	238	38
Marital status		
Never married	22	4
Ever-married	600	96
Religion		
Hinduism	521	84
Islam	51	8
Christianity	19	3
Others	31	5
Education		
No education	03	1
Primary school	18	3
Secondary school	105	17
College	420	67
Professional (MBA, CA, MBBS, etc.)	76	12
Occupation level		
Low	281	45
Medium	112	18
High	229	37

*All percentages rounded to whole numbers

### Presenting complaints/symptoms

The subjects had classic diabetic symptoms. Of the 622 subjects, 273 (44%) had nocturia, 192 (31%) had polyuria, and 145 (23%) had polydypsia. However, 57 (9%) subjects presented with vision impairment **(**Table 2**)**.

**Table 2.** Presenting symptoms of type 2 diabetic subjects from Gujarat, India

Manifestation of symptoms	No.(n=622)	%*
Nocturia	273	44
Polyuria	192	31
Polydipsia	145	23
Vision impairment	57	9
Itching of private parts	50	8
Tingling	136	22
Weight loss	165	27
Weakness	365	59
Leg pain	155	25
Burning micturation	68	11
Skin complaint	59	10
Numbness	39	6
Impotence	28	5

*All percentages rounded to whole numbers

### Behavioural profile

The findings of the study showed that 173 (28%) of the 622 subjects had some form of habits. For example, 56 (9%) were smokers, 121 (20%) were tobacco chewers, and 51 (8%) were consuming alcohol. Most (84%) study subjects reported sedentary lifestyle **(**Table 3**)**.

### Risk profile

A very few (7%) study subjects had good glycaemic control (HbA1c ≤7%). Two-thirds had positive family history of diabetes. The findings showed a mean BMI of 27.06±4.57. According to BMI, only 16% of the sample subjects had normal weight; the majority was either overweight (BMI 23.0-24.9 kg/m^2^) or obese (BMI ≥25 kg/m^2^). Microalbuminuria was found among 10% (n=62) of the subjects. Of the T2DM patients, 78% had dyslipidaemia, and 47% had hypertension **(**Table 3**)**.

**Table 3.** Profile of clinical and other associated factors of type 2 diabetic subjects from Gujarat, India

Characteristics	No. (n=622)	%*
Glycosylated haemoglobin(HbA1c) (mean±SD)	9.02±1.67	
Glycaemic status (%)		
<7 (good control)	46	7
7-8 (sub-optimal control)	159	26
8-9 (sub-optimal control)	163	26
>9 (uncontrolled)	254	41
Family history of diabetes		
Present	417	67
BMI group		
Underweight (<18.5 kg/m^2^)	11	1
Normal (18.5-22.9 kg/m^2^)	99	16
Overweight (23.0-24.9 kg/m^2^)	92	15
Obese (≥25.0 kg/m^2^)	420	68
Microalbuminuria	62	10
Lipid		
Dyslipidaemia	487	78
Hypertension		
Present	289	47
Mode of onset		
Acute	446	72
Sub-acute	145	23
Insidious	31	5
Physical activity		
Sedentary	525	84
Moderate	89	14
Heavy	8	1
Diet control	103	17
Other diabetic treatments (excluding diet)		
Yes	219	35
Past history (of any medical/surgical condition)		
Yes	291	47
Smoking		
Yes	56	9
Years of tobacco smoking		
(mean±SD)	11.46±9.27	
Tobacco chewing		
Yes	121	20
Years of tobacco chewing		
(mean±SD)	12.37±8.61	
Alcohol		
Yes	51	8
Years of alcohol drinking		
(mean±SD)	9.68±7.64	

*All percentages rounded to whole numbers;

SD=Standard deviation

There was a significant (p<0.05) difference between the male and the female subjects with respect to mean age (male=46.93±11.11 years, female=48.95±10.53 years), mean BMI (male=26.47±4.34, female=28.02±4.78), mean waist-circumference (male=93.12±10.47 cm, female=88.96±9.53 cm), mean hip-circumference (male=98.46±8.83 cm, female=101.69±12.52 cm) and mean LDL level (male=119.20±31.23, female=127.73±38.15) (Table 4). BMI was significantly (p<0.001) associated with hypertension (Table 5).

**Table 4.** Characteristics of study population, clinical and laboratory findings by sex among type 2 diabetic subjects from Gujarat, India

Characteristics	Mean±SD		p value
	Male	Female	
Age	46.93±11.11	48.95±10.53	0.026
Body mass index	26.47±4.34	28.02±4.78	<0.001
Waist-circumference (cm)	93.12±10.47	88.96±9.53	<0.001
Hip-circumference (cm)	98.46±8.83	101.69±12.52	<0.001
Blood pressure			
Systolic (mmHg)	128.30±16.43	129.60±17.54	0.354
Diastolic (mmHg)	84.87±9.13	83.41±9.19	0.054
HBA1c	9.07±1.74	8.93±1.55	0.333
Lipid profile			
Cholesterol (mg/dL)	194.82±39.24	201.00±46.98	0.079
HDL (mg/dL)	41.17±5.28	40.91±6.10	0.575
LDL (mg/dL)	119.20±31.23	127.73±38.15	0.003
Triglycerides (mg/dL)	182.49±123.31	169.87±140.23	0.242
VLDL (mg/dL)	36.04±18.46	32.03±12.62	0.004

HDL=High-density lipoprotein;

LDL=Low-density lipoprotein;

SD=Standard deviation;

VLDL=Very low-density lipoprotein

**Table 5.** Factors associated with hypertension among T2DM subjects from Gujarat, India

Factor	Hypertension		χ^2^	p value
	Yes	No		
Age (years)				
Up to 40	67	97	6.86	0.076
41-48	73	97		
49-55	74	76		
>55	75	63		
Body mass index				
≥25 kg/m^2^	222	198	21.25	<0.001
<25 kg/m^2^	67	135		
Physical activity				
Sedentary	246	279	0.21	0.659
Moderate to heavy	43	54		
Lipid profile				
Dyslipidaemia	220	267	1.4	0.242
Normal	69	66		
Family history				
Positive	205	212	3.7	0.054
Negative	84	121		
Glycaemic status (%)				
<7 (good control)	24	22	5.449	0.142
7-8	65	94		
8-9	86	77		
>9	114	140		

## DISCUSSION

Diabetes mellitus is a major public-health problem worldwide. Its prevalence is rising in many parts of the developing world, and India is no exception to this. It will become diabetes capital of the world in near future. Individuals with T2DM are considered on high priority as they are potential candidates for rapid evaluation to prevent and halt the progression of complications.

This study presented observational data from a large number of subjects with diabetes attending the Department of Diabetology, All India Institute of Diabetes and Research and Yash Diabetes Specialties Centre. To the best of our knowledge, no such type of profiles has been reported from Gujarat. Nonetheless, literature on the prevalence of diabetes is available from South and North India (22-24). Our main motivation for this analysis was to obtain the risk profile so that we can prevent or decrease the burden of T2DM in Gujarat.

The present study found that T2DM is a major burden in Gujarat, which is consistent with findings of Simon (4). The main findings of the study were: only 7% of the study population had good glycaemic control (HbA1c ≤7%), 68% of the subjects with T2DM were obese, and BMI was significantly (p<0.001) associated with hypertension. Our study population had a negligible proportion of illiterate T2DM subjects. This finding was expected, given that our sample was drawn from an urban tertiary-care hospital.

Achieving good glycaemic control in diabetic subjects has proven a real challenge to healthcare providers. Studies have documented that self-care among T2DM subjects improved glycaemic control and reduced complications (25-26). In the present study, only 7% of the subjects had good glycaemic control, which is different from various studies. A Swedish survey found that 34% of type 2 diabetes subjects had good glycaemic control (27). Al-Maskari F *et al.* found that 38% of T2DM subjects had good glycaemic control (28), and the study by Al-Kaabi J *et al*. reported that 31% of subjects had good glycaemic control (21). The possible explanation may be that our study sample consisted of newly-diagnosed T2DM subjects drawn from a tertiary-care hospital, and non-adherence to interventions may contribute to uncontrolled glycaemic status (29). These subjects may have T2DM for many years as evident from complications, such as renal dysfunction in 9% and vision impairment in 10% of the T2DM subjects. In the present study, the majority (68%) of the subjects with T2DM were obese. This is consistent with the findings of various studies (30-33). Obesity was also associated with family history of diabetes among the Indian population (34). Dyslipidaemia and hypertension were also related with the family history of diabetes through its link to BMI (32,35). The role of BMI as predictor for hypertension has been previously des-cribed (36), and the present study also confirmed the same among the T2DM subjects. Our study could not demonstrate any significant association among physical activity, dyslipidaemia, and controlled glycaemic status with hypertension among the T2DM subjects. However, the age and family history of diabetes were marginally significant.

Although diet control is the cornerstone in the management of T2DM, only 17% of the study sample was on diet therapy.

### Limitations

Several potential limitations should be considered in interpreting the results of the present study. First, the study is limited by cross-sectional design; so, temporality (cause-and-effect relationships) cannot be established. It can, however, provide a clear snapshot of the current situation and may help improve the management and design of future studies to explore further. Second, this is a hospital-based study in an urban set-up, which may not be representative and applicable to general population. However, this could provide a reasonably precise and reliable estimate of the risk profile of T2DM in Gujarat. Third, we tried to include newly-diagnosed subjects but we are not sure that all subjects are newly diagnosed because we have relied on the subjects.

### Conclusions

The present study directed at providing the profile of the T2DM subjects from Gujarat, India, as an impetus for further exploration of the sociocultural and subject-related factors affecting the outcomes of T2DM care that, in turn, will lead to redefine the diabetes control and preventive strategies. The findings of the study revealed that a high proportion of factors, such as obesity, family history of diabetes, dyslipidaemia, uncontrolled glycaemic status, sedentary lifestyles, and hypertension, were prevalent in the T2DM subjects. The findings also provide an early indication for development of T2DM-related complications.

Based on the findings of the study, we recommend the following steps for the appropriate management of T2DM subject:

- Uncontrolled glycaemic status, dyslipidaemia, and vision impairment should be taken care of by conducting early screening for complications, frequent check-ups, and follow-ups.
- Prevention of T2DM needs to have lifestyle modification interventions, i.e. body-weight control through diet and exercise should be emphasized.

## ACKNOWLEDGEMENTS

The study was supported by All India Institute of Diabetes and Research, Ahmedabad, India. The authors thank all the study subjects and Dr. B.D. Mankad especially for expert opinion. They also thank Dr. Saeed Akhtar, Dr. Prakash J Shah, and Dr. W.Q. Shaikh for reviewing the earlier version of the manuscript. The authors are indebted to Mr. Diwakar Sharma for statistical assistance. They also thank the anonymous referees of the journal for their thoughtful comments that have improved the presentation of the manuscript.
